# Perceptions of Family-Level Social Factors That Influence Health Behaviors in Latinx Adolescents and Young Adults at High Risk for Type 2 Diabetes

**DOI:** 10.3390/children8050406

**Published:** 2021-05-18

**Authors:** Erica G. Soltero, Neeku Navabi, Felipe G. Castro, Stephanie L. Ayers, Jenny Mendez, Deborah I. Thompson, Gabriel Q. Shaibi

**Affiliations:** 1 USDA/ARS Children’s Nutrition Research Center, Department of Pediatrics, Baylor College of Medicine, 1100 Bates St., Houston, TX 77030, USA; dit@bcm.edu; 2 Center for Health Promotion and Disease Prevention, Arizona State University, 500 N. 3rd St., Phoenix, AZ 85004, USA; nnavabi@asu.edu (N.N.); Felipe.Castro@asu.edu (F.G.C.); Stephanie.L.Ayers@asu.edu (S.L.A.); Gabriel.Shaibi@asu.edu (G.Q.S.); 3 Southwest Interdisciplinary Research Center, Arizona State University, 201 N. Central Ave., Phoenix, AZ 85004, USA; 4 Mountain Park Health Center, 6601 W. Thomas Rd., Phoenix, AZ 85033, USA; jemendez@mphc-az.org

**Keywords:** health disparities, Latinx, young adults, adolescents, social support, family dynamics

## Abstract

Given that health behaviors occur within the context of familial social relationships, a deeper understanding of social factors that influence health behaviors in Latinx families is needed to develop more effective diabetes prevention programming. This qualitative study identified perceived family-level social factors that influence health behaviors in Latinx adolescents (12–16 years; *N* = 16) and young adults (18–24 years; *N* = 15) with obesity and explored differences in perceptions across sex and age. Participants completed an in-depth interview that was recorded, transcribed, and coded using thematic content analysis. Emergent themes central to health behaviors included: perceived parental roles and responsibilities, perceived family social support for health behaviors, and familial social relationships. Mom’s role as primary caregiver and dad’s role as a hard worker were seen as barriers to engaging in health behaviors among adolescent females and young adults, males and females. Adolescents perceived receiving more support compared to young adults and males perceived receiving more support compared to females. Health behaviors in both age groups were shaped through early familial social interactions around physical activity. These insights suggest that traditional gender roles, social support, and social interaction around health behaviors are critical components for family-based diabetes prevention programs in high-risk Latinx youth and young adults.

## 1. Introduction

Latino adolescents with obesity have the highest lifetime risk for developing type 2 diabetes (T2D) [1] and exhibit higher rates of prediabetes compared to non-Latino white adolescents (22.6% vs. 11.3%) [2,3]. Adolescence is an important life stage where changes in pubertal insulin resistance, body composition, and decreased physical activity can impact future health trajectories [4]. Prediabetes in adolescence significantly increases diabetes risk as youth transition into young adulthood [5,6] and it is estimated that 40% of young adults with prediabetes convert to overt T2D within five years [7,8]. To address growing T2D disparities, there is a need for targeted, evidence-based diabetes prevention strategies, particularly during the transitional life stage of adolescence to young adulthood. However, few diabetes prevention programs have been developed for Latinx adolescents and young adults [9,10].

### Familial Influences in Youth Health-Related Behaviors

Physical activity interventions are a first-line approach to diabetes prevention among youth [9]. The most effective disease prevention strategies for addressing health disparities in minority youth to date are those that utilize a family-based approach and leverage familial relationships and social processes for health and health behavior change [11,12]. The Ecodevelopmental Model provides a framework for mapping complex interactions across cultural, social, and familial environmental systemic domains on individual-level health factors including diet, physical activity, and disease outcomes. In addition, this model maps the influence of these complex interactions on individual health across life stages, such as the transition from adolescence to adulthood [13]. At the macro-level, social and cultural determinants shape parents’ health-related beliefs, practices, and their sense of control over their own health and health behaviors, including physical activity [14,15]. In turn, these macro-level factors influence the child’s health as they shape the health-related family social processes shared between a parent and child, which ultimately influence the child’s individual health behaviors and outcomes [16,17]. Thus, the family social environment has emerged as a critical micro-level environment for health promotion and disease prevention [16,17,18,19,20].

To date, a large body of evidence has demonstrated the importance of family social support [21,22,23] and parenting practices for improving health behaviors, such as physical activity [20,24,25]. While social support and certain health-related parenting practices have been associated with diet and physical activity in younger Latinx children, less is known regarding these family-level social factors and their influence on these health behaviors in Latinx adolescents and young adults [19,20,26,27]. Among Latinx families, social factors that influence health and health behaviors are transmitted culturally within the context of family relationships. For example, familism, or familismo, is a cultural value that is salient among Latinx families and refers to strong family connectedness, bonding, and includes health-related customs regarding health behaviors [28,29]. To develop effective family-based behavior change strategies for Latinx youth, there is a need to increase our understanding of Latinx familial social factors and how they are operationalized in the sociocultural context of Latinx families [15,17].

The primary purpose of this qualitative study was to identify and compare perceived family-level social factors that influence health behaviors in Latinx adolescents and young adults with obesity. Perceived family-level social factors like social support can differ by sex [30,31,32] and there are also sex differences in perceived support from one’s mother vs. one’s father [33]. Furthermore, perceptions in family-level social factors may also change as youth transition from adolescence to young adulthood [34]. Thus, the secondary aim of this study was to explore differences in perceived family-level social factors across sex and age. This aim represents a unique opportunity to illuminate differences in perceptions of family-level factors that influence health behaviors across two critical life periods. Findings will yield insights that can guide the development of prevention strategies in these understudied populations.

## 2. Materials and Methods

### 2.1. Participants

This qualitative study builds on existing work from two National Institutes of Health (NIH)-funded projects aimed at integrating and operationalizing social and cultural determinants of health into lifestyle interventions to address T2D disparities in Latinx youth and young adults. Participants were recruited through a vast network of community stakeholders, pediatric clinics, and local Spanish-language media. Interested participants were screened using the following inclusion criteria: (1) self-identification as Latino (a), (2) age 12 to 16 years for adolescents and 18–22 years for young adults at enrollment, and (3) obesity, defined as BMI ≥ 95th percentile for age and sex or BMI ≥ 30 kg/m^2^. BMI was confirmed using measures of height and weight. Exclusion criteria included the following: (1) taking medication(s) or diagnosed with a condition or injury that influences metabolism, physical activity, or cognition, (2) pregnant, or (3) diagnosed with T2D.

### 2.2. Interview Procedures

Youth and young adults participated in an in-depth interview between August 2018 and August 2019. Two trained interviewers conducted all interviews in-person in a private conference room at Arizona State University or in a private room within a public library conveniently located in the participant’s neighborhood. All interviews were conducted in English and were digitally recorded. Participants were compensated $25 for their time. All study procedures and materials were approved by the Institutional Review Board at Arizona State University and participants provided written informed consent or assent prior to their participation.

### 2.3. Interview Guide

A semi-structured interview guide was developed by the investigative team in collaboration with community stakeholders. Interview questions were informed by the Expanded Ecodevelopmental Model [13]. The final interview guide included 10 open-ended questions; however, to fulfill the purpose of the current study, only qualitative data from the questions presented in Table 1 are included in the current analyses. Prompts and probes were used as needed to elicit information for each question.

### 2.4. Qualitative Data Analysis

Interviews were professionally transcribed verbatim through a transcription service. Transcripts were coded by two trained coders using thematic content analysis in the qualitative analysis software program NVivo (Version 12.5, QSR International, 2019) [35]. In the first step, coders independently read each transcript within the software program. Based on study objectives and open-ended responses to interview questions, codes were developed using repeated ideas and important quotes regarding family-level social factors that influence health behaviors to independently generate an initial list of codes within Nvivo. Once the first reading was completed, coders met to compare and contrast codes within their codebooks. Codes were either confirmed, modified, or rejected, to yield one guiding codebook that contained a list of codes and code definitions. Using the updated codebook, coders followed the same process of independently applying codes to interview transcripts to complete three additional independent readings of the transcripts [36]. Coding stopped once study objectives were ‘saturated,’ indicating that coders did not identify additional codes and no new information was identified, signaling that data collection was complete. Following the coding process, codes were then organized into groups of related family-level social factors that informed emerging themes and associated subthemes [37]. Themes and subthemes were then compared across age and sex.

## 3. Results

**Figure 1:**
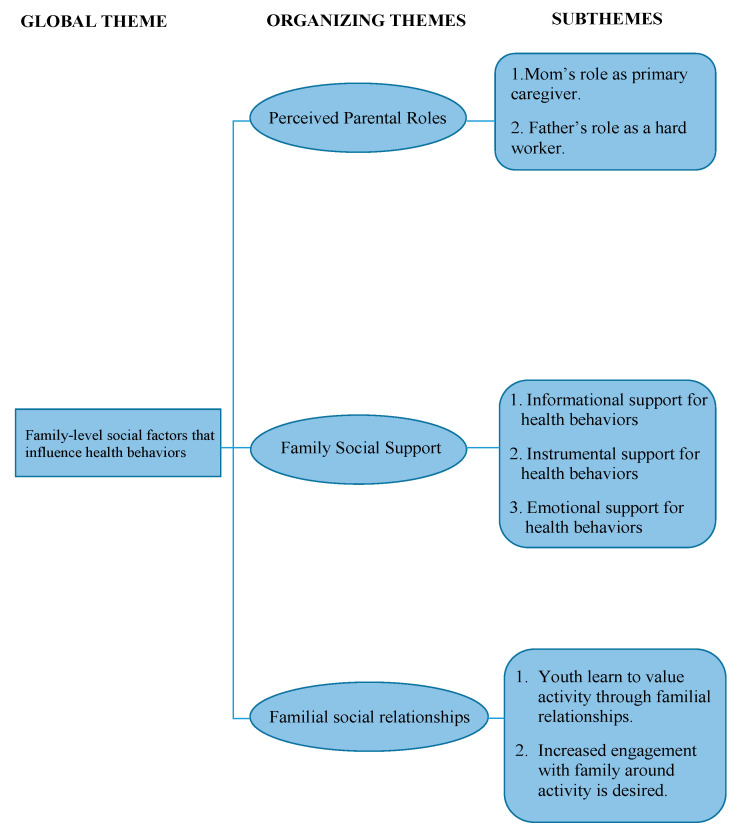
Identified themes and subthemes.

Interviews were conducted among 16 adolescents and 15 young adults and each interview lasted about an average of 30 min (22–47 min). Table 2 compares participant characteristics for adolescents (*N* = 16) and young adults (*N* = 15). The majority of youth (63%) and young adults (73%) reported living in a two-parent household. All adolescents were currently enrolled in junior high or high school. Nearly 70% of young adults reported that they had completed high school. Most adults (73%) were employed full time, with about 20% currently attending a two-year or four-year college or university. Half of the young adults (56%) reported that they had access to health insurance and only one young adult was a parent. Figure 1 presents the thematic network for this study and provides an overview of the emergent, organizing themes and subthemes that support the study’s global theme of family-level social factors that influence health behaviors. The quotes below represent exemplar quotes that support themes and subthemes. To provide context, quotes are identified by age group (adolescent or young adult) and sex.

### 3.1. Theme 1. The Influence of Perceived Parental Roles and Responsibilities on Health Behaviors

When asked about parental influence on health behaviors, both youth and young adults responded by describing the perceived roles and responsibilities that their parents have within the family. Moms were often described as caregivers (Subtheme 1) and dads were typically described as hard workers (Subtheme 2). Participants went on to describe the perceived impact of these roles and responsibilities on health behaviors.

#### 3.1.1. Subtheme 1: Mom’s Role as Primary Caregiver Influences Health Behaviors

Most adolescents (88%) felt that their mothers influenced their health through caregiving. Youth discussed that their mothers express care and concern for their health through grocery shopping, cooking, and providing transportation to doctor appointments or sports practices. Mothers were often described as selfless, and youth expressed appreciation for the way their mom prioritizes the care of the family.

*“I’d say my mom definitely is a big one. She would always take us hiking, or ... She would try to do things with us.”*

—Adolescent female

*“…having all these kids and then every day waking up early, making my dad food. At some point we had three uncles living with us, she’d wake up earlier just to get them food and then me and my sister would wake up and she gives us food and then we go to school. When we get back, she already has food prepared. Mothers are one of the hardest workers I think we have.”*

—Adolescent male

However, over half of the adolescent female participants (63%) also went on to express feelings of frustration because their mother’s caregiving responsibilities often limited opportunities for them to engage with their mother around health or in general. This was not discussed among adolescent male participants.

*“My mom would either have to work late or work on the weekends...I barely even sometimes get to talk to her cause she has to take care of the kids, or run some errands, and clean the house.”*

—Adolescent female

*“My mom, when we were in the program [health promotion program], she would go with us, and she would take us…but I feel like she has a lot of responsibilities around the house, and like, well, now that she has the baby, it’s different, ‘cause when she had the baby, we had to start going by ourselves to the program.”*

—Adolescent female

Although to a lesser extent, many young adults (67%) also discussed their mom’s caregiving activities, like grocery shopping and cooking, as important influences on their health behaviors.

*“She usually is the one that does the grocery shopping so we eat healthier. Yeah, she’s the cook.”*

—Young adult female

In contrast to adolescent females, young adults did not discuss mom’s caregiving as a factor that limits engagement. However, three young adult females shared that they are taking on more caregiving responsibilities within the family as they transition into young adulthood. They discussed their new role as a caregiver as a factor that limits opportunities for them to engage in health behaviors like physical activity.

*“Just putting more work on me, taking care of my brother and my sister or, “Go do this for me,” or, “Go do that for me.” That doesn’t give me time to do a bunch of what I would like to do, but just the fact that I have to help them [parents] out with most of the chores at the house, the driving here and there… It doesn’t give me enough time to do activities that I would like.”*

—Young adult female

#### 3.1.2. Subtheme 2: Dad’s Role as a Hard Worker Influences Health Behaviors

When discussing dad’s influence on their health behaviors, about half (63%) of all adolescents immediately began discussing their dad’s long working hours and rigorous work schedule. Most adolescent males (75%) were more likely to express that they perceived their dad as supportive of health behaviors, but that he was often not home or too tired to engage in health behaviors like play time or sports play. However, adolescent males were more likely to be supportive of their dad’s role as a hard worker. In fact, some shared that they looked up to their dad for the way he provides for his family and were motivated by his work ethic.

*“…he [dad] still does a pretty laborious job, like construction, he still does that to feed everyone in the house. And seeing that makes me want to work hard as well…instead of him always taking care of us, I could take care of them [participant’s family] as well.”*

—Adolescent male

In contrast, while some adolescent females reported that their dad was an influence on health behaviors within the household, over half of adolescent females (63%) expressed that they have limited interactions with their dad around health behaviors because of dad’s work schedule. Adolescent females were also more likely to describe themselves as having a more distant relationship with their father.

*“Well, my dad, I feel because he’s not really around the house, because he works a lot, and he gets home tired. I don’t spend a lot of time with him. My brother is maybe the one that spends a little bit more time with him, because either they do yard work, or something together…”*

—Adolescent female

*“My dad works from 8:00 in the morning to 6:00… I don’t really care because I’m not as close to him, because I barely see him, and he doesn’t get me.”*

—Adolescent female

Similarly, about 40% of young adults mentioned their father’s role as a hard worker when discussing their dad’s influence on health behaviors. Similar to female adolescents, young adults expressed that while their dad does influence health behaviors in the household, interactions with dad around health behaviors are limited due to his rigorous work schedule.

*“My Dad, always on his feet, never stops… He just runs the restaurant, non-stop…For sure my dad can work and at times when he gets home, he might want to rest, most of the time. That’s when, he’s just exhausted…”*

—Young adult male

*“He [participant’s dad] just works and goes to sleep. I don’t really see him throughout the week except for Saturday and Sunday. Throughout the week he works. He leaves early in the morning. He comes back mid-afternoon. He stays sleeping. I get home like at six. I don’t really see him.”*

—Young adult female

### 3.2. Theme 2: Perceived Family Social Support for Health Behaviors

As participants continued to discuss their physical activity habits and familial influences on their health behaviors, they discussed occasions where they received social support for health behaviors from parents, siblings, and extended family members such as grandparents or cousins. Based on definitions found in the literature [21,22], coders categorized the type of social support received from family as either informational, instrumental, or emotional social support. These codes were reflected in the codebook and used to identify the type and source of the support as well as differences in perceived support across sex and age.

#### 3.2.1. Subtheme 1: Informational Social Support for Health Behaviors

Informational support includes knowledge, advice, or suggestions on healthy eating and physical activity behaviors [21,22]. About half of adolescents (44%) discussed receiving informational support for health behaviors from family. This included only three adolescent male participants who discussed receiving informational support from their dad or siblings.

*“Also, my little brother, he would always tell me, ‘cause over there at school, there’s really bad breakfast so he would say, “Choose the best cereal and stuff. If there’s none that are good, then you better not get some…” On Fridays they always do cinnamon rolls and donuts or pop-tarts, he says, “Just get cereal” and I said, “Okay.”
*

—Adolescent male

In contrast, half of adolescent female participants discussed receiving informational support and were more likely to receive this type of support from their mom.

*
“My mom was always telling me to eat my fruits and vegetables, and she’s making us take vitamins…* *She always makes our food, so she puts like vegetables in there… She’s always telling me to exercise with her.”*

—Adolescent female

In contrast, almost all young adult male participants (72%) discussed receiving informational support, whereas informational support was only discussed by one young adult female participant. For young adult males, this type of support primarily came from mom.

*“My Mama. She’s the one who always like tells me to get up and do something [physically active].”*

—Young adult male

#### 3.2.2. Subtheme 2: Instrumental Social Support for Health Behaviors

Instrumental support from parents includes providing resources, modeling, or engaging in health behaviors with one’s child, and was one of the most common types of support discussed among adolescent participants [21,22]. Almost all adolescent male participants (87.5%) discussed receiving instrumental support from a broad range of sources, including mom, dad, and other family members. The majority of adolescent female participants (75%) also shared instances where they received instrumental support for health behaviors; however, this support was most likely to come from their mom.

*“We [participant and dad] play soccer together once or twice a week.”*

—Adolescent male

*“I mean, my mom will go to the grocery store, and she lets me pick out the things that I wanna buy, like when it comes to like fruits or vegetables…or sometimes, she helps me prepare different meals. Definitely, my mom is one of the main people that helps me and encourages me to eat better and to exercise.”*

—Adolescent female

Similar to adolescents, instrumental support was the most common form of support discussed among young adults. Over half of the young adult males (57%) discussed instances where they received instrumental support, and similar to adolescent males, this support was more likely to come from a variety of family members. Over half of the young adult females (62.5%) also reported receiving instrumental support; however, in contrast to adolescent females, they were more likely to receive this type of support from their siblings, followed by their mom. There was no mention of female young adults receiving instrumental support from dad.

*“She [participant’s mom] goes to the gym four times a week, so, yeah. She is a good example.”*

—Young adult male

*“Usually we [participant and participant’s mom] take walks every afternoon. Probably that’s like the only time that I feel active…We just go to the park, walk around with the dogs.”*

—Young adult female

#### 3.2.3. Subtheme 3: Emotional Social Support for Health Behaviors

Emotional support for health behaviors is defined as providing praise or encouragement for behavior change [21,22,23]. Half of the adolescent male participants discussed receiving emotional support for health behaviors. Among adolescent male participants, this type of support was primarily received from their mom. About 40% of adolescent female participants discussed receiving emotional support for health behaviors, and similar to adolescent males, this support was primarily received from mom. No adolescent participant shared an occasion where they received emotional support from their dad.

*“Recently, she [participant’s mom] talked to me, she’s like, “I know you don’t like being the way how you are,” Cause I tell her I don’t feel good, cause you can feel the weight difference. So yeah. And she was like, “You know, you should do it [lose the weight]. I know you can do it.”*

—Adolescent male

*“Because she [participant’s mom] cares about me, and she cares about my health and my goals…Like, I tell her my goals, and she wants to help me to get where I wanna be.”*

—Adolescent female

Similar to adolescent males, about half of young adult males also discussed receiving emotional support for health behaviors. However, in contrast to adolescent males, this support was more likely to come from siblings and extended family members like cousins. Similar to adolescent females, about 40% of young adult females received emotional support primarily from their mom.

*“For sure, they [participant’s family members] tell me, as well, ‘Keep it up, you’re doing good’…I have their support, as well. So that’s a good thing that I get to have to motivate me to go to the gym every day.”*

—Young adult male

*“I think it’s just my mom, she tells me like, when I was working out, she’s like ‘oh I like seeing you be active, I like seeing you drink more water’, and stuff like that.”*

—Young adult female

**Figure 2:**
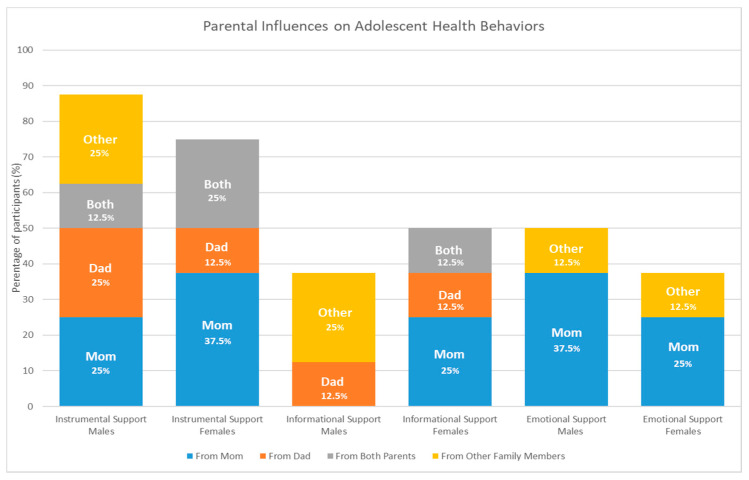
Adolescent perceived family social support for health behaviors.

**Figure 3:**
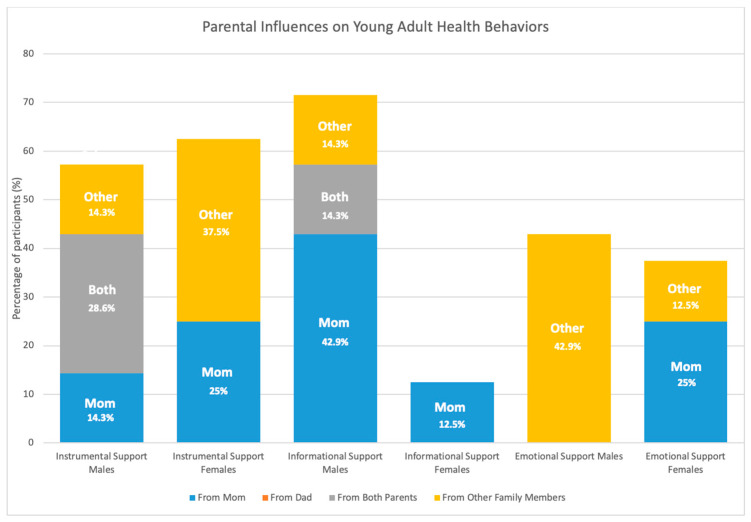
Young adult perceived family social support on health behaviors.

Qualitative data summarizing differences in perceived social support and source of support across sex and age were quantified and presented for adolescents in Figure 2 and young adults in Figure 3.

### 3.3. Theme 3: Familial Social Relationships Are Central to Health Behaviors

To learn more about perceptions of health behaviors in the context of Latinx families, youth and young adults were asked the following question: ‘Does your family think being active is important?’ Participants were then prompted to explain how they knew whether their families valued being active, which led to the emergence of subthemes.

#### 3.3.1. Subtheme 1: The Value of Activity Is Learned through Familial Relationships

Most adolescents (88%) learned that their families valued physical activity by observing parents and family members engage in physical activity. These responses indicate that familial social processes like behavioral modeling and co-participation in physical activity communicate the value placed on physical activity within the family context.

*“My dad, he thinks that it [physical activity] was so important that he made a soccer team with me...He’s the coach.”*

—Adolescent male

*“I think they see it already. I think they’re always active, and they motivate me to be active with them…so then I’m going to be active too…and I get two things at the same time, spending time and then being active”*

—Adolescent female

In contrast to adolescents, only about 40% of young adults were more likely to respond that while they think that family members know that being active is important, they often do not engage in physical activity due to time limitations. In discussing time as a barrier to being active, some young adults reflected on how they used to engage in health behaviors with their family more frequently when they were younger; however, this changed when they transitioned into young adulthood.

*“Now that we’re a little older, we’re not all together… My brother goes to school and my sister goes to school. My brother gets home at like six because of his sports. My sister gets home at like five because she stays after school for something. Then my dad, he gets home from work but he goes straight to sleep… so we’re not always together during the weekdays. I think the only day that we are all together is on a Sunday. Changing that to being together the whole week and trying to figure out, “Hey, what are we going to eat all together? What should we make, something different, something a little more healthier?” Just not doing our own thing all the time.”*

—Young adult female

*“For sure, when I was younger, I was way more active with my family. Walking around, going places and things like that….”*

—Young adult male

#### 3.3.2. Subtheme 2: Increased Engagement with Family around Activity Is Desired

In the follow-up discussion to this question, participants in both age groups expressed a desire to engage with family in activity more frequently and reiterated that time is often a barrier:

*“I wish we could just have more time to be with each other... a little less time working and more time focusing on what activity we should do together as a family.”*

—Adolescent female

*“I wish we would do more active things. I wish I could go with them to the zoo, walk around, hang out at the mall. It’s just our conflicting lives and schedules.”*

—Young adult male

## 4. Discussion

By the year 2050, the Latinx population will represent 29% of the U.S. population [38] and it is estimated that 50% of Latinx youth will develop T2D in their lifetime [1]. Developing effective diabetes prevention strategies for this rapidly growing population is essential for addressing diabetes disparities and reducing the public health burden of this costly disease [3]. Family-based T2D prevention interventions that are designed to address family-level social factors that influence T2D-related health behaviors and outcomes are needed to develop more effective diabetes prevention programming among Hispanic youth and young adults. This requires a deeper level of understanding of these family-level factors, thus the primary aim of this cross-sectional, qualitative study was to provide insights and contextual information on perceived family-level social factors that influence health behaviors in Latinx adolescents and young adults with obesity. A secondary aim was to explore differences in perceived family-level social factors across gender and age.

### 4.1. Influence of Traditional Gender Roles

Adolescents and young adults perceived their moms as the primary caregivers in the home. Compared to males, many adolescent females felt that their mom’s caregiving responsibilities limited their ability to engage in activities together. This is consistent with previous studies that have reported that Latinx mothers traditionally assume the role of caregivers and that this role has deep cultural significance [39,40,41]. Other studies have found that because caregiving is an important cultural role, some Hispanic mothers do not view themselves as having the time for leisure physical activities for themselves or with family [42]. A few young adult women, particularly if they were the firstborn or oldest child, described taking on more family caregiving responsibilities as they transitioned into young adulthood, which they recognized as a factor that limited their time for activity. From a cultural perspective, the caregiver role is traditionally nurtured and assigned to the oldest daughter and there is an expectation that she will contribute more to family caregiving as she gets older [40]. Among both adolescents and young adults, dads were described as hard workers. While adolescent males embraced this role, and some were motivated by this role, adolescent females and young adults perceived dad’s work schedule as a factor that limited opportunities to engage with him around health behaviors. This is consistent with other studies that have found that Latinx fathers experience high levels of stress and fatigue from physically demanding occupations, which can serve as barriers to engaging in activities with their children [43,44,45]. In the U.S., Latinx men are more likely to work manual labor jobs, work non-traditional hours, and have multiple physically demanding jobs [43,46]. The role of ‘hard worker’ and ‘family provider’ has deep cultural significance as a strong work ethic and providing for one’s family are important cultural values among many Latinx men [47,48]. These findings demonstrate that strong beliefs about cultural and traditional mother and father roles within the Latinx family differentially influence health behaviors across age and sex. Our findings also indicate that as youth transition into young adulthood, particularly females, they may start to take on these traditional roles, further influencing their health behaviors.

### 4.2. Effects of Social Support

Mothers were perceived as providing the most support across all three social support subtypes, including instrumental, informational, and emotional support. This is perhaps not surprising given their role as the primary caregiver. Dads were more likely to provide instrumental support to their children, followed by informational support, and there were no reports of dads offering emotional support. This is consistent with other reports demonstrating that dads are more likely to offer instrumental support in the form of co-participation with children in physical activities compared to mothers [49]. Adolescent males discussed receiving instrumental support, from a variety of family members, and emotional support, primarily from their mom, at a higher frequency than adolescent females, who perceived receiving more informational support. Compared to adolescent males, young adult males perceived receiving more informational support. In contrast, young adult females perceived receiving less informational support than adolescent females. Young adults from both sexes reported receiving high levels of instrumental support and this was more likely to come from both parents for males and from mom and other family members for females. Emotional support among young adults was more likely to come from siblings and cousins for young adult males and from mom and siblings for young adult females. Previous studies have shown that parents are more likely to provide instrumental support for physical activity to male children compared to female children [50,51] and that female children perceive less support for physical activity from their parents, who are more protective and restrictive towards daughters (i.e., daughters are less likely to be allowed to play outside) [52]. This study confirms these findings by demonstrating that in the context of Latinx families, mothers and fathers provide different types of support and they provide different types of support to daughters and sons. Young adults in this sample were more likely to receive support from siblings, cousins, and grandparents, suggesting that family members outside of their parents play a bigger role in providing social support from health behaviors among this age group.

### 4.3. Effects of Familial Social Relationships

The final emergent theme observed in this study demonstrated that both youth and young adults learn to value physical activity by engaging with family through activity and desire more opportunities to spend time and to be active as a family. This finding supports the call for family-based diabetes prevention strategies [17,53]. Engaging parents in prevention efforts is often considered important because youth are not yet fully autonomous [54]. However, beyond the issue of autonomy, these findings suggest that a family-based approach is culturally relevant and desired by Latinx adolescents and young adults. Compared to adolescents, young adults spoke of the decrease in family physical activity as they transitioned into young adulthood. Interestingly, most Latinx young adults (73%) in this study still lived in the same household as their parents. In the U.S., Latinx young adults are more likely to remain and return to their parental home compared to non-Hispanic white young adults [55]. These findings demonstrate that engaging youth early in physical activity experiences with family is important for demonstrating the importance of being active. While less time may be spent in physical activity with family as youth transition into young adulthood, the family social environment and structure may still have an important influence on health behaviors.

### 4.4. Directions for Future Research with Latinx Families

This study represents a first step in gaining insight from Latinx adolescents and young adults on how to adapt and refine family-based health promotion and disease prevention strategies. To start, these findings suggest that family-based interventions should assess variations in adherence to traditional cultural roles and responsibilities and discuss familial health behaviors within this context. It is important for future studies to identify ways in which these roles can be leveraged for health promotion within the family in a manner that respects the cultural significance of traditional male and female roles reflected in the qualitative data presented in this study. For example, mothers should be empowered to promote health behaviors in their family through caregiving and role modeling. Additionally, family members should be encouraged to support mothers in making healthy changes, as lack of family support for healthy changes in food consumption is often a barrier that can discourage Latina mothers from introducing new foods and activities [56,57]. Despite reporting limited interactions with their dads due to his work schedule, it was clear from participants that dads are still sources of support for health behaviors. Studies have demonstrated that because of this role as the family provider and head of the household, dads have a unique influence on the health of their children and are important agents of behavior change [45,58,59]. Future studies should examine how to leverage Latinx fathers’ status as the family leader for health promotion and disease prevention opportunities. Studies that have focused on Latinx men have found that connecting engagement in health behaviors to their values of being a provider and spending quality time with their family increases program participation [43,44]. More research is needed to understand how beliefs about traditional gender roles influence health behaviors and disease outcomes, and future culturally grounded diabetes prevention strategies should facilitate discussions among families on roles and responsibilities that may be critical for health promotion and disease prevention efforts across both youth and young adult age groups [60,61].

Given the importance of a supportive family social environment to improving health behaviors [17,34,62], there is a need for family-based interventions to inform both parents of the importance of providing social support to their children [63]. This includes raising awareness that boys and girls may need different types of support from both parents [21]. Given that parental support for health behaviors typically declines during adolescence [34], parents should be encouraged to continue to provide support for health behaviors as their child transitions into young adulthood. Future studies should aim to identify the type of support that is needed across gender and age groups from both mom and dad to improve health behaviors in Latinx youth and young adults [51]. Studies focused on young adults may also need to move beyond dyadic relationships with parents to include siblings and extended family members.

In this study, social interactions with family members shaped beliefs on the value of physical activity within the familial context. As youth transition into adulthood, lack of time emerged as a barrier to being active with family; however, engagement with family around activity was still desired among young adults. Family-based diabetes prevention strategies have shown promise among Latinx youth [60,64], including disease prevention strategies that have focused on engaging Latinx dads [65]. However, more research is needed to develop and test family-based strategies that engage and evaluate parents as co-participants and educate parents on the importance of modeling, co-participating, and providing support for health behaviors. For strategies that focus specifically on Latinx young adults, studies may need to adopt a broader definition of family given that siblings and cousins emerged as important familial social relationships for health behaviors.

## 5. Conclusions

Given that health behaviors are developed in the context of the family’s ecologic and social environment, there is a need to identify family-level social factors that influence health behaviors and disease outcomes in Latinx youth and young adults. This qualitative study on family-level social factors and social processes found that cultural and traditional parental roles and responsibilities are perceived as having an influence on health behaviors. Mom’s role as the primary caregiver was an important influence on health behaviors; however, caregiving responsibilities presented barriers to spending time with mom for adolescent females, and young adult females discussed transitioning into this caregiving role, which limited opportunities to be active. As the provider for the family, dad’s work long, hard hours, which was admired by some youth, particularly adolescent males, but seen as a barrier to engaging around health behaviors among adolescent females and young adults. These findings suggest the need to acknowledge and discuss cultural gender roles in future health promotion programs. This study also revealed differences in perceived social support for health behaviors across sex and age groups, with adolescents perceiving more support compared to young adults and males perceiving more support compared to females. Future interventions should reinforce the importance of both parents providing support to both male and female children. Lastly, this study revealed that physical activity behaviors in both age groups were shaped by familial social interactions and both adolescents and young adults desired more engagement with family around physical activity. This evidence supports previous calls for more family-based disease prevention programming. Future qualitative research is needed to expand upon this work to provide further insight on familial factors and social processes that can be leveraged for health promotion and disease prevention among high-risk Latinx families.

## Figures and Tables

**Table 1:** Interview questions.

Tell me about the last time that you felt like you were being active? Describe where you were, who you were with, and what you were doing.
2. What are some ways that your family influences your health behaviors?
3. Does your family think being active is important?

**Table 2:** Participant characteristics.

Variable	Adolescent (*N* = 16)	Young Adults (*N* = 15)
Sex (*N*)		
Male	8	7
Female	8	8
Age (years)	14.6 ± 1.5	20.7 ± 1.1
Mean BMI (kg/m^2^)	37.2 ± 5.6	41.8 ± 9.0
Living Status (%)		
Two-parent household	62.5	73.3
Female-headed household	18.8	6.7
Separated parents	12.5	6.7
Blended Family	6.3	13.3

## Data Availability

The data presented in this study are available upon request from the corresponding author. The data are not publicly available due to privacy and ethical concerns.
